# RTP801/REDD1: a stress coping regulator that turns into a troublemaker in neurodegenerative disorders

**DOI:** 10.3389/fncel.2014.00313

**Published:** 2014-10-02

**Authors:** Mercè Canal, Joan Romaní-Aumedes, Núria Martín-Flores, Víctor Pérez-Fernández, Cristina Malagelada

**Affiliations:** Department of Pathological Anatomy, Pharmacology and Microbiology, Faculty of Medicine, University of BarcelonaBarcelona, Catalonia, Spain

**Keywords:** RTP801, REDD1, mTOR, Akt, stress, neurodegeneration, neuron, Parkinson’s disease

## Abstract

Mechanistic target of Rapamycin (mTOR) pathway regulates essential processes directed to preserve cellular homeostasis, such as cell growth, proliferation, survival, protein synthesis and autophagy. Importantly, mTOR pathway deregulation has been related to many diseases. Indeed, it has become a hallmark in neurodegenerative disorders, since a fine-tuned regulation of mTOR activities is crucial for neuron function and survival. RTP801/REDD1/Dig2 has become one of the most puzzling regulators of mTOR. Although the mechanism is not completely understood, RTP801 inactivates mTOR and Akt via the tuberous sclerosis complex (TSC1/TSC2) in many cellular contexts. Intriguingly, RTP801 protects dividing cells from hypoxia or H_2_O_2_-induced apoptosis, while it sensitizes differentiated cells to stress. Based on experimental models of Parkinson’s disease (PD), it has been proposed that at early stages of the disease, stress-induced RTP801 upregulation contributes to mTOR repression, in an attempt to maintain cell function and viability. However, if RTP801 elevation is sustained, it leads to neuron cell death by a sequential inhibition of mTOR and Akt. Here, we will review RTP801 deregulation of mTOR in a context of PD and other neurodegenerative disorders.

## RTP801 overview

RTP801 (also known as REDD1 or Dig2) is a protein encoded by the stress responsive gene DNA-damage-inducible transcript 4 (*DDIT4*). It was initially identified and cloned in 2002 by two different groups simultaneously.

Shoshani et al. (2002) screened for hypoxia-regulated genes in rat C6 glioma cells and they identified a highly up-regulated gene responsive to HIF-1, involved in the regulation of cellular reactive oxygen species (ROS). It was designated *RTP801* (Shoshani et al., 2002).

Concurrently, Ellisen et al. (2002) cloned a gene induced after DNA damage and during embryogenesis in a p53 and p63 dependent manner. This gene was involved in the regulation of ROS and was alternatively named *REDD1*, for regulated in development and DNA damage responses one (Ellisen et al., 2002).

Later in 2003, Wang et al. discovered Dig2 (for dexamethasone-induced gene 2), the mouse homolog of *RTP801/REDD1* in an oligonucleotide microarray analysis from dexamethasone-treated murine lymphoma T cells (Wang et al., 2003).

Further studies displayed *DDIT4* as a rapidly upregulated gene under multiple cellular stresses, such as heat shock (Wang et al., 2003), ionizing radiation (Ellisen et al., 2002), hypoxia (Shoshani et al., 2002; Brugarolas et al., 2004) and energy depletion (Sofer et al., 2005). Moreover other chemical molecules also upregulated *DDIT4* expression, such as dopaminergic neurotoxins 6-hydroxydopamine, MPTP/MPP+ and rotenone (Malagelada et al., 2006), endoplasmatic reticulum (ER) stress inducers tunicamycin and thapsigargin (Wang et al., 2003; Whitney et al., 2009), DNA damage agent etoposide (Wang et al., 2003) and arsenite (Lin et al., 2005b).

RTP801, as a 232 aminoacids protein, is ubiquitously expressed at low levels in numerous human adult tissues (Shoshani et al., 2002). RTP801 localizes in the cytoplasm, the nucleus (Ellisen et al., 2002; Lin et al., 2005b; Michel et al., 2014) and in the membranes (DeYoung et al., 2008; Michel et al., 2014). Besides, a small fraction of RTP801 was detected in the mitochondria in HEK293T cells (Horak et al., 2010) and in *RGC*-*5* retinal ganglion cell line (del Olmo-Aguado et al., 2013).

There is a related human transcript called RTP801L (RTP801-like) or REDD2 that displays ~50% sequence identity to RTP801 and has similar functions (Ellisen et al., 2002; Corradetti et al., 2005).

Besides humans, RTP801 is also present in other organisms such as rat, mouse and *Xenopus*. In *Drosophila* it has two related orthologs called Scylla and Charybdis (Reiling and Hafen, 2004).

No functional motifs or structural domains could be identified from RTP801 amino acid sequence analysis and, to date, the entire crystal structure has not been solved. Indeed, only one group crystallized a segment containing aminoacids 89–226 with a deletion of the hydrophobic region ^200^FLPGF^204^ of the human RTP801 protein (Vega-Rubin-de-Celis et al., 2010). Their work has given new insights into RTP801 structure. They found that RTP801 presents a unique topology characterized by a two-layered α/β sandwich with a psi-loop motif. Furthermore, a surface patch formed by highly conserved residues was found to be critical for its function. It is formed by two separated regions, ^138^EPCG^141^ and ^218^KKKLYSSE^225^, that are contiguous in the three-dimensional structure (Vega-Rubin-de-Celis et al., 2010). Importantly, the stretch of three lysines ^218^KKK^220^ is necessary for RTP801 to localize in both mitochondria (Horak et al., 2010) and plasma membrane (Michel et al., 2014).

The key function of RTP801 is its ability to inactivate mTOR (Brugarolas et al., 2004; Corradetti et al., 2005), a master regulator kinase that integrates extracellular signals with intracellular responses to nutrients, growth factors or stress. Indeed, mTOR regulation has a crucial role in development, cancer or in neural survival and plasticity (Hoeffer and Klann, 2010; Laplante and Sabatini, 2012).

RTP801 has a dual role depending on the cellular context, meaning that in proliferating non differentiated cells, RTP801 is anti-apoptotic, and in non-dividing differentiated cells like neurons, RTP801 is pro-apoptotic (Shoshani et al., 2002; Malagelada et al., 2006). This dual function could be nicely observed in a study of rat cortical neurogenesis, where RTP801 controlled neuroprogenitors proliferation and neuronal differentiation. In cortical neuroprogenitors RTP801 was elevated without being toxic. On the contrary, newborn and mature neurons showed lower levels of RTP801. Indeed, if RTP801 elevation was sustained in these differentiating neurons it became pro-apoptotic (Malagelada et al., 2011). How RTP801 can trigger these dual actions based on the cell context is not completely understood and requires further investigation.

In the last decade the role of mTOR in neural cells has become very relevant. Indeed, in the nervous system mTOR controls crucial processes such as protein translation, long-lasting synaptic plasticity and survival via Akt (Tang et al., 2002; Cammalleri et al., 2003; Malagelada et al., 2008). Evidence suggests that mTOR deregulation is involved in neurodegeneration, and therefore the role of RTP801 has emerged as an object of study. In this sense, it will be crucial to understand the fine balance between RTP801 as a stress-coping protein and RTP801 as a pro-apoptotic effector in neurological disorders. Understanding these complex mechanisms will help to design successful therapeutic strategies to halt or, at least, delay neurodegeneration.

## How is RTP801 upregulated?

Previous studies suggest that RTP801 toxicity in neurons is proportional to its protein levels (Malagelada et al., 2010; Ota et al., 2014). RTP801 protein increase can be the end point of two different processes: (1) as a result of gene activation by cellular stress (Ryu et al., 2005; Malagelada et al., 2006); and (2) a defective RTP801 degradation (Romaní-Aumedes et al., 2014). Here we will describe the transcription factors responsible for *DDIT4* gene induction, the microRNAs (miRNAs) that regulate its translation and the post-translational events in charge of regulating RTP801 protein levels.

### RTP801 transcriptional regulation

The variety of transcription factors able to induce *DDIT4* gene expression in response to different stressors illustrates the complexity of its regulation. In fact, one feature of the regulation of RTP801*/DDIT4* is its rapidity, crucial to activate the coping mechanisms of the cell in response to the hostile environment. For example, hypoxia upregulates RTP801 expression via HIF-1, since the *DDIT4* gene contains a HRE (hypoxia-response element) in the promoter (Shoshani et al., 2002; Brugarolas et al., 2004). Another hypoxia-mimetic agent, cobalt chloride (CoCl_2_), needs co-activation of HIF-1 and Sp1 to induce RTP801 (Jin et al., 2007).

Deoxyribonucleic acid (DNA) damaging agents, including ionizing radiation and the DNA alkylating agent methyl methane sulfonate (MMS) also boosted RTP801 expression (Ellisen et al., 2002; Lin et al., 2005a). Ionizing radiation induced RTP801 in a p53-dependent manner in mouse embryonic fibroblasts (MEFs; Ellisen et al., 2002). DNA-damage-inducible transcript 4 transcription was also enhanced by MMS in human keratinocytes via Elk-1 and CCAAT/enhancer-binding protein (C/EBP) in a p53-independent manner (Lin et al., 2005a). Furthermore, RTP801 has also been identified as a transcription target of Elk-1 and C/EBP in response to arsenic-induced ROS (Lin et al., 2005b).

Endoplasmatic reticulum (ER) stress caused by tunicamycin or thapsigargin upregulated RTP801 via activating transcriptional factor 4 (ATF4; Jin et al., 2009; Whitney et al., 2009). ATF4 was also identified as a transcription factor for RTP801 in response to oxidative stress induced by hydrogen peroxide (Jin et al., 2009). Interestingly, ATF4 has a protective role in cellular models of Parkinson’s disease (PD) by modulating the levels of the E3 ligase parkin (Sun et al., 2013).

Other transcription factors have been described for *DDIT4* gene regulation like the nuclear factor of activated T-cell c3 (NFAT c3; Zhou et al., 2012) or PLZF in spermatogonial progenitors (Hobbs et al., 2010).

It is noteworthy that all these stressors, via different transcription factors, elevate RTP801 with a common objective to inactivate mTOR. This common response to stressors corroborates the complexity of the integration of the stress signals to modulate mTOR effectively.

Many other stress responsive genes with pro-apoptotic functions are also upregulated in parallel with DDIT4. However, due to space limitations, they will not be reviewed in this text (reviewed in Fulda et al., 2010).

### RTP801 translational regulation

MicroRNA are negative regulators of gene expression and can function as tumor suppressors or oncogenes. To date, at least three miRNAs have been described as regulators of RTP801/REDD1 expression in a context of tumorigenesis. MiR-495 regulates breast cancer stem cells proliferation and hypoxia resistance by regulating RTP801 expression (Hwang-Verslues et al., 2011). Another miRNA, the miR-221, stimulates hepatocarcinogenesis by downregulating RTP801 expression (Pineau et al., 2010). Furthermore, Micro-RNA30c down-regulates REDD1 expression in human hematopoietic and osteoblast cells after gamma-irradiation (Li et al., 2012).

To our knowledge, no miRNA that modulates RTP801 expression in a context of neurodegeneration has been described. In a near future, miRNAs along with the long non-coding RNAs may have a relevant impact in the regulation of RTP801 levels and function.

### RTP801 post-translational regulation

Post-translational modifications like phosphorylation, acetylation, ubiquitination or myristoylation, have an important impact in protein stability, function and cellular localization. Indeed, cellular stresses can also affect turnover of many proteins, including RTP801.

Apart from its rapid gene induction under stress, RTP801 proteostasis will also determine its stability, and therefore, its regulatory function towards mTOR.

Related to that, RTP801 mRNA (Wang et al., 2003) and RTP801 protein (Kimball et al., 2008; Katiyar et al., 2009; Malagelada et al., 2010) half-lives are significantly short, revealing that RTP801 is an extremely unstable protein with a fine-tuned post-translational regulation.

One of the modifications that will lead to a rapid protein turnover is ubiquitination. In fact, RTP801 is poly-ubiquitinated and targeted for the ubiquitin-proteasome system (UPS; Katiyar et al., 2009; Romaní-Aumedes et al., 2014).

To date, only three E3 ring ligases have been identified to poly-ubiquitinate RTP801. The first one described was CUL4A-DDB1-ROC1-β-TRCP E3 ligase complex. The complex ubiquitinated RTP801 and targeted it for proteasomal degradation, in a GSK3β-phosphorylation-dependent manner (Katiyar et al., 2009).

The second E3 ubiquitin ligase for RTP801 was HUWE1/MULE that modulated RTP801 protein levels although this regulation seemed to be UPS-independent (Tan and Hagen, 2013).

So far, the role of both ligases in regulating RTP801 in neurodegenerative disorders has not been elucidated.

In our recent work, we found that parkin RING E3 ligase poly-ubiquitinates RTP801 to mediate its UPS degradation. Based on the results obtained in cellular and animal models and in samples from human parkin mutant carriers, we proposed that RTP801 elevation due to parkin loss-of-function in both parkin mutants and in idiopathic PD might contribute importantly to neurodegeneration (Romaní-Aumedes et al., 2014).

In a near future, other ligases will eventually be proved to ubiquitinate RTP801, due to its central role in regulating mTOR.

## RTP801 inactivates mTOR via tuberous sclerosis complex

Under stress conditions downregulation of mTOR activity caused by hypoxia, energy stress or exposure to dopaminergic neurotoxins required the expression of RTP801 and an intact TSC1/TSC2 tumor suppressor complex (Brugarolas et al., 2004; Sofer et al., 2005; Malagelada et al., 2006). Interestingly, despite the clear necessity of the intact TSC1/2 complex for RTP801 to downregulate mTOR, RTP801 does not seem to interact physically with either TSC1 or TSC2 (Vega-Rubin-de-Celis et al., 2010).

Tuberous sclerosis complex (TSC) is a heterodimer formed by two proteins, the tuberous sclerosis tumor suppressors TSC1 and TSC2. TSC2 has a catalytic function as a GTPase activating protein (GAP), and it acts toward the small GTPase Rheb, an upstream positive mTOR regulator (Inoki et al., 2003; Tee et al., 2003). TSC2 can be phosphorylated by several kinases in response to upstream signals, and they will modify its regulation towards mTORC1 (reviewed in Ma and Blenis, 2009). Akt can phosphorylate TSC2 in response to growth factors (Inoki et al., 2002). This event is thought to favor TSC2 binding to protein 14-3-3, instead to TSC1, leading to TSC2 inhibition, to finally activate mTORC1 (Cai et al., 2006).

Regarding the modulatory role of 14-3-3 towards TSC2, DeYoung et al. (2008) proposed a molecular mechanism by which RTP801 regulates TSC1/2-mTOR signaling involving 14-3-3. Other studies also reported interaction between RTP801 and 14-3-3 proteins by co-immunoprecipitation experiments (Favier et al., 2010; Hernández et al., 2011; Pieri et al., 2014).

However, this RTP801 direct binding to 14-3-3 has been questioned by others (Vega-Rubin-de-Celis et al., 2010). The supposed 14-3-3 binding motif in RTP801 (^133^RLAYSEP^139^) is not conserved within species and the crystallized RTP801 structure analysis does not reveal any established mode for 14-3-3 binding. Thus, the inhibitory mechanisms of RTP801 towards mTOR need to be further investigated.

## RTP801 upregulation in neurodegeneration

The etiology of PD and many other neurodegenerative disorders involves both environmental factors and genetic predisposition. Indeed, exposure to several environmental toxins such as pesticides and metals has been implicated in its pathogenesis (Migliore and Coppede, 2009; Cannon and Greenamyre, 2011; Baltazar et al., 2014).

Arsenic is a heavy metal that has been linked to neurotoxicity and carcinogenesis in humans by a mechanism involving ROS production (reviewed in Qian et al., 2003). Interestingly, arsenite induced RTP801 transcription (Lin et al., 2005b).

Moreover, extensively used dithiocarbamate pesticides Maneb (MB) and Mancozeb (MZ) induced DNA damage and elevated RTP801 mRNA and protein expression (Cheng et al., 2014). The toxicity mechanism of these pesticides has been linked to NF-κB activation, revealing a cross-talk between RTP801 and NF-κB (Cheng et al., 2014).

In line with this, RTP801 was also identified as an amyloid-β-peptide (Aβ) responsive gene. Amyloid-beta is a neurotoxic molecule and the main component of senile plaques in Alzheimer’s disease (AD; Kim et al., 2003).

Dopaminergic neurotoxins also upregulated RTP801 in cellular and animal models. Specifically, RTP801 was upregulated at both transcriptional and protein level in neuronal PC12 cells treated with PD mimetic toxins 6-OHDA, MPP^+^ and rotenone, and it was also induced in neurons of MPTP-treated mice and in Substantia Nigra pars compacta (SNpc) degenerating neurons of PD patients. Furthermore, RTP801 knockdown with short hairpin RNAs (shRNAs) protected the cultures from PD mimetic toxins (Malagelada et al., 2006).

Ubiquitin-proteasome system deregulation has been implicated in neurodegenerative disorders (Keller et al., 2000; Jana et al., 2001; McNaught et al., 2003). Accumulations of misfolded or aggregated proteins may hamper cellular functions and eventually lead to neuronal death (reviewed in Tanaka and Matsuda, 2014). RTP801 brief protein half-life suggests that UPS malfunction would affect RTP801 protein levels. Therefore it is logical to suggest that a proper RTP801 degradation might be crucial for neuron function and survival.

Thus, chronic and progressive RTP801 elevation could become a hallmark of neurodegenerative disorders due to a combination of stress-induced gene activation and UPS malfunction.

## The role of RTP801 in neurodegenerative disorders

RTP801 behaves like many other stress-induced genes, where a small increase is beneficial but a chronic and sustained increase is detrimental for the neuron. Interestingly, since RTP801 is able to inactivate protein translation, via mTOR, and survival, via Akt (Malagelada et al., 2006, 2008, 2010), its pro-apoptotic role may be relevant to other neurodegenerative diseases.

In PD, RTP801 elevation was initially identified in cellular and animal models of the disease. These results were further confirmed in nigral neurons from both idiopathic PD and mutant parkin human brains, meaning that RTP801 may have a relevant role in the disease itself (Malagelada et al., 2006).

Since RTP801 elevation is necessary and sufficient to trigger neuronal death, it is logical to think that the upregulation in the SN from PD human brains might be detrimental for the nigral neurons. Interestingly RTP801 induces cell death through a mechanism involving TSC1/2 and mTOR repression (Malagelada et al., 2006). Indeed, RTP801 repression of mTOR led also to the suppression of the phosphorylation of the neuronal survival kinase Akt (Malagelada et al., 2008), reviewed in (Greene et al., 2011). This negative regulation of Akt was also observed in nigral neurons from PD human brains.

**Figure 1:**
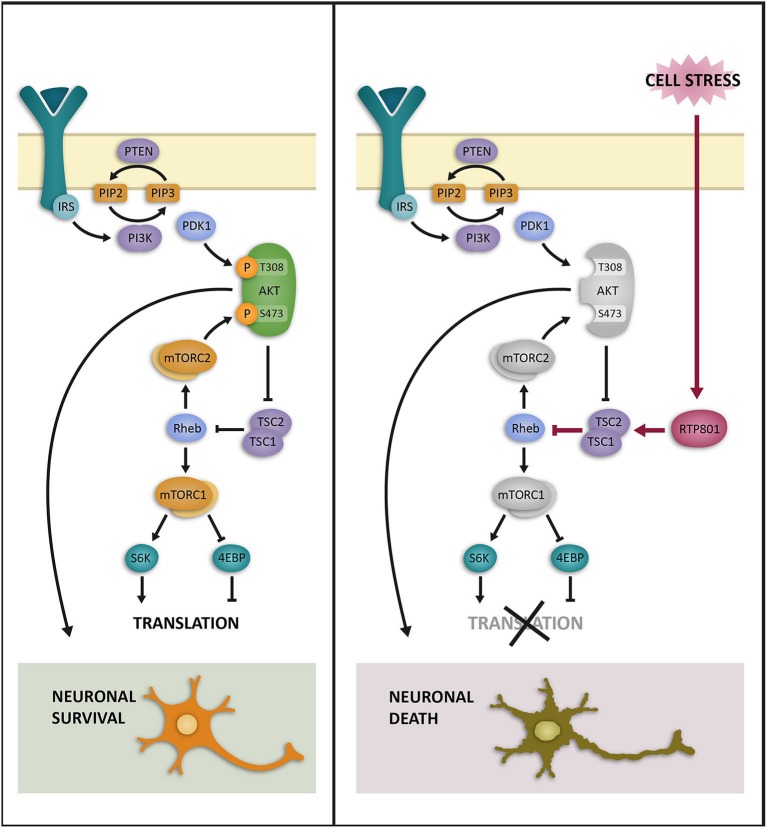
**Schematic representation of the hypothesized regulation of mTOR/Akt by RTP801 in neurons**. In physiological conditions the gene and the protein levels of RTP801 are low; mTOR is active and promotes protein translation (mTORC1) and Akt phosphorylation at Ser473 residue (mTORC2). These signals mediate neuronal survival (left panel). However, when neurons are under stress, RTP801 is induced at gene and protein levels, and promotes mTORC1 and mTORC2 inhibition through TSC1-TSC2 complex and Rheb protein. These events result in protein translation inhibition and prevent Akt phosphorylation at residues Ser473 and, consequently, at Thr308. If this mTOR/Akt repression is sustained over time neuron function is impaired and leads to neuron death (right panel). *Illustration by Olivares-Boldú L*.

Based on these results, we proposed a mechanism to explain RTP801 contribution to neurodegeneration in PD, where low levels of RTP801 would help neurons to cope with stress, but if this inactivation were sustained in time, it would impair neuronal function and survival. At the latest stage, and when the critical threshold of mTOR/Akt inactivation is surpassed, neuronal death will occur (Malagelada et al., 2006, 2008; see Figure 1). This proposed mechanism is supported by the observation of elevated RTP801 and diminished Akt phosphorylation in nigral neurons of PD brains (Malagelada et al., 2006; Romaní-Aumedes et al., 2014).

To extend this working model, one possibility would be that at early stages of many neurodegenerative disorders, RTP801 induction by cellular stresses would contribute to mTOR depression in an attempt to preserve neuron function and viability. However, at more advanced disease stages, when RTP801 upregulation is prolonged in time, it can eventually promote neurodegeneration and neuronal death through mTOR and pro-survival kinase Akt inactivation, as it has been proposed in the PD studies.

In AD an elevation of both RTP801/REDD1 gene and protein was observed in lymphocytes from AD patients compared to age-matched controls. Damjanac et al. (2009) showed that Double-stranded RNA-dependent protein kinase (PKR), a cognitive decline biomarker with an impairing protein translation function, by phosphorylating p53, activated REDD1 gene transcription. It could be interesting to explore whether this mechanism can be observed also in hippocampal neurons or in AD murine models.

Recently, RT801 has been determined crucial for stress-mediated synaptic loss and depressive behavior. Chronic unpredictable stress increased RTP801 mRNA and protein levels in rat prefrontal cortex (PFC). This RTP801 upregulation was coincident with a reduced phosphorylation of mTORC1/2 signaling targets S6K, 4EBP1, and Akt. In agreement, RTP801 was elevated in postmortem PFC of patients with major depressive disorder compared to psychiatrically healthy controls. RTP801 knockout mice were resilient to the synaptic and behavior deficits caused by stress, while RTP801 overexpression in rat PFC was sufficient to promote neuronal atrophy and depressive behavior (Ota et al., 2014).

Further investigations are needed to elucidate the role of RTP801 in many other neurological disorders. It will be interesting to explore whether RTP801 levels are upregulated and whether they correlate with the degree of mTOR/Akt repression. Also, it will be important to determine whether RTP801 is promoting neuron death through the same mechanism proposed as in PD models.

## RTP801 as a potential therapeutic target in neurological disorders

Neurodegenerative disorders are characterized by neuronal death of specific subpopulations, such as loss of SN dopaminergic neurons in PD (reviewed in Dauer and Przedborski, 2003). However, current PD therapies ameliorate symptoms but do not prevent neuronal death. Thus, it is essential to investigate the mechanisms that underlie neuronal death and identify targets involved in the pathophysiology (reviewed in Levy et al., 2009). Based on several studies, RTP801 could become one such potential therapeutic target (Brafman et al., 2004; Malagelada et al., 2010; Tarazi et al., 2014).

RTP801 promotes cell death by sequentially inactivating mTOR and Akt (Malagelada et al., 2006, 2008). Thus, compounds that can restrain RTP801 expression or modulate the mTOR/Akt pathway may become therapeutic and delay neurodegeneration and neuronal cell death in PD (reviewed in Tarazi et al., 2014).

One such compound is 8-methyl-6-phenoxy-2-(tetra-hydropyran-4-ylamino)pyrido[2,3-d]pyrimidin-7-one (FLZ), a synthetic squamoside derivative from a Chinese herb that protected dopaminergic neurons from apoptosis triggered by MPP^+^ and 6-OHDA (Zhang et al., 2007a,b). Interestingly, FLZ neuroprotective actions in PD involved activation of Akt/mTOR signaling pathway and inhibition of RTP801 expression (Bao et al., 2012, 2014).

Another promising compound is rapamycin, an allosteric inhibitor of some but not all mTOR activities. Rapamycin conferred neuroprotection in both cellular and animal models of PD (Tain et al., 2009; Dehay et al., 2010; Malagelada et al., 2010). The results obtained support the hypothesis in which rapamycin blocks RTP801 translation and, as a consequence, it mitigates mTOR repression leading to Akt phosphorylation maintenance at a site critical for its pro-survival activity (Malagelada et al., 2010). In contrast, Torin 1, an inhibitor of all mTOR actions since it blocks the ATP-binding site, was not protective and promoted Akt dephosphorylation and neuron death. So, rapamycin protection derives from its partial suppression of certain mTOR actions due to its allosteric properties (Malagelada et al., 2010).

Nonetheless, further investigations are required to evaluate the potential therapeutic role of these two agents in PD.

In animal and cellular models of cerebral ischemia, Ligustilide, a major active agent of Radix Angelicae Sinensis, the root of a chinese herb called Danngui, is neuroprotective by inhibiting RTP801 expression, in addition to promote Erythropoietin transcription via extracellular-signal-regulated kinases (ERK) signaling pathway (Wu et al., 2011).

The most advanced example of RTP801 as a therapeutic target is found in retinopathies. RTP801 has an important role in the pathogenesis of retinopathies, since the absence of RTP801 expression in a mouse model attenuated the development of the disease (Brafman et al., 2004). PF-04523655, a 19-ribonucleotide siRNA designated to inhibit RTP801 transcription is currently in clinical trials for retinopathy treatment (Lee et al., 2012; Nguyen et al., 2012a,b; Rittenhouse et al., 2014).

In summary, RTP801 is one clear example of a protein that is upregulated to cope with cellular stress although its sustained progressive elevation leads to neuron degeneration and death. RTP801 progressive elevation and its inhibitory function towards pro-survival kinases mTOR and Akt could explain its role in several neurodegenerative diseases.

## Conflict of interest statement

The authors declare that the research was conducted in the absence of any commercial or financial relationships that could be construed as a potential conflict of interest.
